# Successes and challenges in extracting information from DICOM image databases for audit and research

**DOI:** 10.1259/bjr.20230104

**Published:** 2023-09-12

**Authors:** Alistair Mackenzie, Emma Lewis, John Loveland

**Affiliations:** 1 NCCPM, Royal Surrey NHS Foundation Trust, Guildford, United Kingdom; 2 Scientific Computing, Royal Surrey NHS Foundation Trust, Guildford, United Kingdom; 3 CVSSP, University of Surrey, Guildford, United Kingdom

## Abstract

In radiography, much valuable associated data (metadata) is generated during image acquisition. The current setup of picture archive and communication systems (PACS) can make extraction of this metadata difficult, especially as it is typically stored with the image. The aim of this work is to examine the current challenges in extracting image metadata and to discuss the potential benefits of using this rich information. This work focuses on breast screening, though the conclusions are applicable to other modalities.

The data stored in PACS contain information, currently underutilised, and is of great benefit for auditing and improving imaging and radiographic practice. From the literature, we present examples of the potential clinical benefit such as audits of dose, and radiographic practice, as well as more advanced research highlighting the effects of radiographic practice, *e.g*. cancer detection rates affected by imaging technology.

This review considers the challenges in extracting data, namely,

**•** The search tools for data on most PACS are inadequate being both time-consuming and limited in elements that can be searched.

**•** Security and information governance considerations

**•** Anonymisation of data if required

**•** Data curation

The review describes some solutions that have been successfully implemented.

**•** Retrospective extraction: direct query on PACS

**•** Extracting data prospectively

**•** Use of structured reports

**•** Use of trusted research environments

Ultimately, the data access process will be made easier by inclusion during PACS procurement. Auditing data from PACS can be used to improve quality of imaging and workflow, all of which will be a clinical benefit to patients.

## Introduction

Practices and technology in imaging centres have continued to advance since becoming fully digital, with improved quality of systems,^1^ new image processing techniques^2,3^ and additional imaging modes.^4^ To assess and optimise the impact of these developments requires ready access to clinical and imaging data. Anecdotal evidence from imaging centres suggests information that is often not readily available to staff even to undertake simple audits. Restrictions can be due to picture archiving and communication system (PACS) setup or access limits imposed by administrators. However, there are many potential benefits from access to the large amount of data on PACS.^5–8^ The UK government recognises^9^ uses of NHS clinical data for research and improving patient care:

1. For the direct care of individuals.
2. To improve population health through the proactive targeting of services.
3. For the planning and improvement of services.
4. For the research and innovation that will power new medical treatments.

Digital information can be held within a range of radiology systems: the Hospital Information System (HIS) and Radiology Information system (RIS) contain patient and diagnosis/treatment information. The use of international standards^10^ such as HL7 and DICOM (Digital Imaging and Communications in Medicine) for transfer of clinical and image data respectively has eased access to data. However, manufacturers of radiology systems interpret and implement these standards differently, particularly in emerging technologies such as tomosynthesis, increasing the complexity of data extraction.

DICOM is primarily a standard for image transfer, in which image data and header are stored together. The header contains “metadata”, information about the patient, image acquisition, and image display parameters.^11^ Although, the metadata for each image typically represents an extremely small proportion of the total file size, it generally cannot be retrieved without the corresponding image pixel-data, considerably adding to retrieval time. The DICOM standard (part 4)^10^ only specifies a small number of data tags that are required to be searched on and does not specify that the metadata be available by itself.

This review aims to discuss the potential benefits of using this rich information and examine current challenges in extracting image metadata from PACS. This narrative review was based on the personal experience of the authors in extracting data securely from a variety of systems and literature searches, using a variety of sources. The intention is to consider applications of data and data access for local and national audits of radiographic practice, as well as research. Although we focus on breast screening, many of the issues and solutions covered here apply to other radiology modalities.

### Applications of data extraction

This section highlights the value of extracting and analysing metadata in a range of applications; Table 1 shows a summary of published examples.

**Table 1.** Summary of studies analysing extracted data

Use case	References	Geography (local, national)	Number of cases	Manufacturers (single/multi)
Clinical audit	Holland et al^12^ Hill et al^13^ Mercer et al^14^ Waade et al^15^ Moshina et al^16^	National 2 centres 3 centres National Local	57,179 1291 975 17,951 25,286	Single Multi Multi Multi Single
Cancer detection	Seradour et al^17^ Chiarelli et al^18^ Bosmans et al^19^ Evans et al^20^ Weigel et al^21^	Regional Regional National Regional Regional	162,257 688,418 189,953 50,000 740,728	Multi Multi Multi Multi Multi
Dose audit	Loveland et al 2022^22^ Weir et al 2021^23^	National National	51,753 n/s^*a*^ (25,927 images)	Multi Multi

a n/s not stated.

### Workflow optimisation

Interrogating DICOM data enables detailed examination of workflow. The length of time for a screening visit is important; in the UK the aim is for a visit-length as short as 5 min, as any increases may cause problems in the service. The DICOM header contains timestamps showing image acquisition date-times. These can be used to track time between examinations, or to examine throughput for examination types.^24–26^ Of course, this method has limitations as it does not give reasons why an appointment lasts longer or that attendees are late or do not attend and does not account for difficult cases.

Talati et al^25^ stated that it is generally easier to get this information from a RIS system, but the timestamps recorded in the DICOM header-data provide more accurate results. From these data, they were able to show that the average MRI examination time was shorter with more experienced radiographers. An audit of the workload of radiographers can give an indication of the time taken between screens as well as between individual images and may allow optimisation of processes. Kathiravelu et al^26^ describes a method of data collection directly from DICOM, which has advantages over RIS as it avoids errors due to transfer to another system.

### Audit of clinical practice

Data can also be used for clinical audit to ensure consistent clinical practice among staff and assure that equipment is functioning correctly. Mercer et al^14^ and Waade et al^15^ performed audits of compression force in England and Norway respectively. Both audits identified variability, finding that the difference between screening centres was larger than between mammographic systems. This is useful to understand as compression affects both image quality and likelihood of cancer detection; *e.g.* Holland et al^12^ and Hill et al^13^ examined compression pressure. They noted that sensitivity, based on occurrence of interval cancers, was lowest at lowest pressure. A similar audit performed for breast tomosynthesis showed a weaker relationship between cancer detection and compression.^16^

### Use of metadata in AI

There has been substantial development of AI applications for mammography, including image-reading, but extending to triage, risk prediction and clinical audit amongst others.^27^ The ability to query based on a wide range of data-points is vital to ensure that training, test and validation data sets are representative of the population, or to support the creation of enriched data sets.

For image-reading, the AI may use additional clinical data or information from the image metadata as well as the image itself. There is variability on how many and which data-points are used, although most AI models are reliant on interpreting the image alone. It is clear, however, that there is useful information within the metadata. Litchfield et al^28^ show that there is a relationship between image appearance and some metadata fields; they also showed that combined information was useful to identify studies from the same client acquired on different days.

Interestingly, Zufiria et al^29^ noted that caution is required in the use of metadata during model training, since it can introduce additional bias, *e.g*. if a particular system is used more commonly within an assessment unit, then the AI model may learn that that type of system is associated with more cancers.

### Audit of clinical outcomes

Undertaking audit of clinical outcomes through the use of imaging metadata is also possible, though more challenging. Audits can examine the effect of technology and reader-experience on clinical outcomes such as cancer detection and recall rates. There are two main methods of investigating efficacy of readers, namely the use of enriched test-sets, with enhanced number of cancers, and clinical audit. There is some evidence of a relationship^30^ between clinical outcomes and performance on test sets; however, the use of limited test sets does not tell the whole story. Data analysis of clinical outcomes in large-scale, multicentre studies has reported differences in cancer detection between technologies.^17–21^ These studies showed the real-life limitations of computed radiography and confirmed that full-field digital mammography should be used. A larger-scale national data set may enable identification of differences beyond those seen in these studies; however, confounding factors such as population differences, image reader variability, and imaging systems and setups used make this challenging. Nevertheless, the ability to investigate the effect of different systems including image processing could be a powerful tool. Observer studies have shown that variations in image processing can have an effect on cancer detection^31,32^ but this has not been assessed in the clinical environment. To obtain the sensitivity necessary to detect such differences clinically may require multicentre, or even national, data collection.

### Dose audit

Data collection methods have variously used manual collection, automatic reading of header-data of small batches of images and fully integrated dose-management systems. Dose-management systems enable collection of very large numbers of cases with relative ease.^33,34^ Dose audits can be undertaken simply using the value for dose in the DICOM header, however these values may be inaccurate, so ideally full radiographic data and compressed breast thickness data should be extracted and dose calculated.^35^

There have been a series of national dose surveys in the UK. Loveland et al^22^ reflects that whilst some screening units have the ability to extract a large amount of data easily, for some even extracting a relatively small amount of data was cumbersome and time-consuming. The survey found variation of average breast dose by compressed breast thickness very similar to that shown in previous UK surveys, but noted that the average compressed breast thickness for mediolateral oblique views has increased from 54.3 mm in 1997–1998^36^ to 62.4 mm in 2016–2019. Such data can be used to set diagnostic reference levels, *e.g.* Weir et al^23^ for different breast thicknesses.

### Challenges in implementing data extraction

The World Health Organisation (WHO) found in a 2016 survey of European member states that the primary barriers to adopting big data for health were privacy concerns, security concerns, and insufficient integration and standardisation.^37^ Additional complications arise from the inherently sensitive nature of medical data; in most countries the use of such data is subject to strict ethical and legal restrictions. Practical issues can make accessing data difficult. Most PACS restrict the fields to be searched. Furthermore, typically it is only possible to view the metadata one image at a time, and there is usually no option to retrieve it for further analysis or processing.^38^ In effect the data are inaccessible, indeed the majority of information stored in PACS archives is never accessed again by healthcare providers.^39^

Several third-party tools are available for accessing DICOM metadata. However, these are typically marketed for tracking radiation doses, with an emphasis on radiographic exposure factors. Commercial dose-management software can cost up to £20k per hospital per year^40^ and still require significant staff resources for setup and maintenance. Free, open-source options are available,^40^ however these usually require in-house high-level technical skill as well as co-operation with the local IT department. Most local NHS IT departments adopt a stringent approach to IT security, often whitelisting applications to minimise risk, and discouraging or prohibiting use of open-source software. Whilst such a whitelisting policy is understandable, and considered by some to be a gold-standard approach,^41^ we speculate that it may encourage the use of unofficial devices and web-based tools, thus increasing the risk of data and cyber-security breaches. Indeed, a gov.uk report provides advice on the use of open source software and its use for use in service analysis and research.^9,42^

The DICOM standard has continued to evolve and manufacturers have sometimes interpreted the standard in different ways. Further challenges obstruct standardisation of descriptive fields such as study, examination, and protocol names. Although the DICOM standard (Part 3, chapter 8) includes options for coded entry data,^10^ often these tags remain as free text fields, which vary widely even within the same organisation. As new equipment is installed in hospitals and as technology develops, workflows and scan-protocols evolve. For many hospitals, this process is unlikely to have been overseen with a coherent approach. The result can be a confusingly large number of protocol names, which are often difficult to relate to common clinical exams. Even basic information such as the institution name can be variable on different equipment. Santos et al^39^ found that the same institution appeared within their PACS system with several different names. 20 years ago, there was a 15% error rate on the “Body Part Examined” DICOM tag^43^ and it is unlikely to have improved; it is still common to find non-compliant and incorrect DICOM metadata. The DICOM standard itself now supports a structured report format, which provides a subset of highly coded information from the metadata; these have the scope to help further standardise the reporting and storage of clinical reporting and results. Aiello et al^44^ reported a number of barriers to its implementation, primarily due to resistance to moving from current reporting methods but also due to the diluting of focus on the image to the report and may increase the reading time and risk of errors.

The lack of a centralised system can lead to substantial duplication of effort with many smaller groups having to undertake similar work to establish their local systems. Even within the same organisation, different types of data are stored on different software systems, often with no straightforward way to link them. The problem is even more pronounced nationally, with many different systems storing nominally the same input data in different information in different formats.

### Data collection pipeline

**Figure 1.**
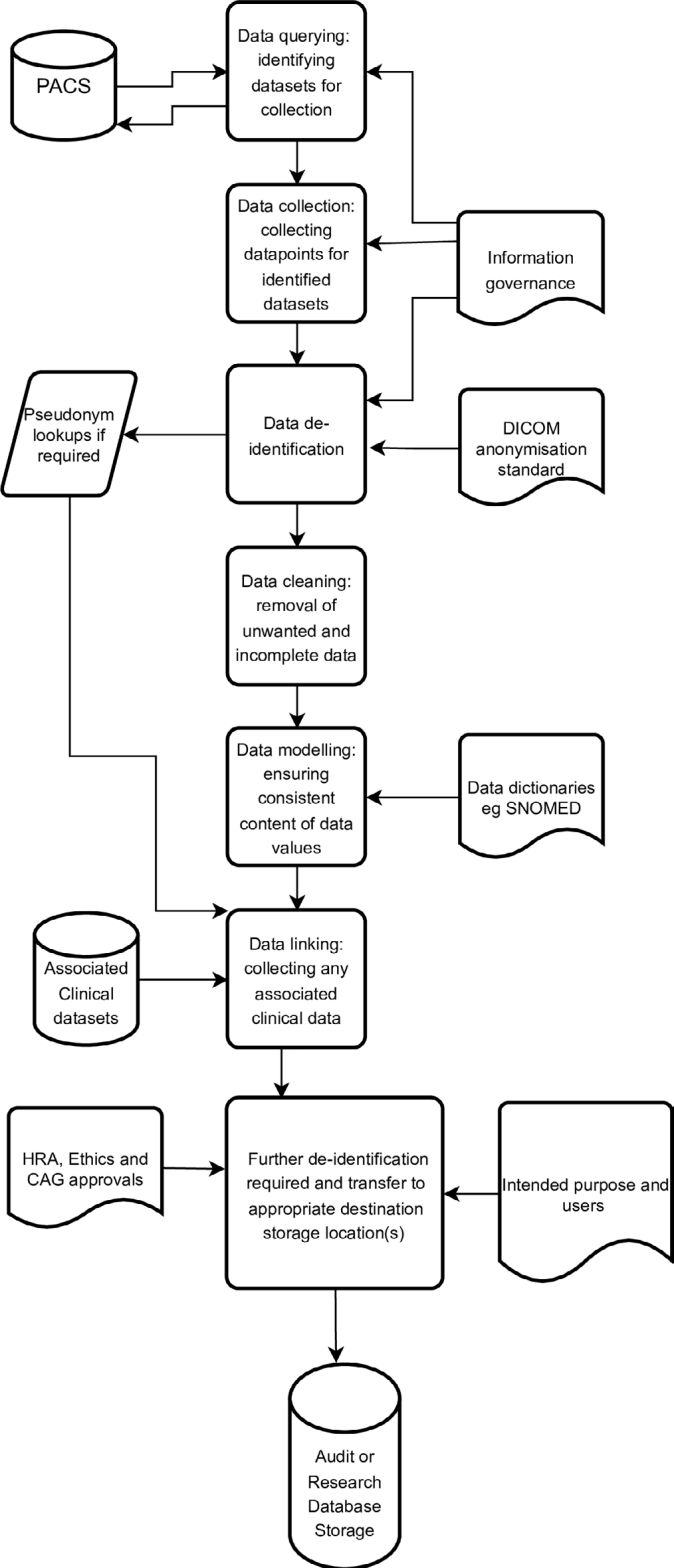
Suggested image metadata collection pipeline. DICOM, Digital Imaging and Communications in Medicine; PACS, picture archiving and communication system.

The task of extracting information from potentially unstructured, linked clinical and imaging data requires multiple, interrelated stages as shown in the pipeline in Figure 1. The pipeline incorporates case selection, image/data retrieval, anonymisation/de-identification, data-cleaning, data curation and finally data storage. Important issues surrounding these stages and proposed approaches are discussed below.

### Data access

A PACS client provides a user interface for manual query and selection of required patient data based on a limited number of filters. However, limitations on the set of header data-points that can be searched in accordance with the DICOM standard severely restricts selection of relevant metadata data sets. For large-scale retrieval of data sets, PACS systems can be configured to auto-forward (“push”) data to an external DICOM application for onwards extraction and storage of metadata. Such external applications are typically configurable with selection and extraction criteria based on DICOM attributes. In both approaches, data are transferred using DICOM storage and communication protocols, and, as noted earlier, limitations around interoperability of DICOM components remain, particularly in regards to metadata.^45^

### Information governance

It is vital that access to personal data is essential and justifiable, hence data may be pseudonymised or anonymised during extraction. The main purposes for acquiring and processing clinical and imaging data are: (i) as part of the clinical pathway, (ii) for use in clinical audit and (iii) for research or patient benefit. Depending on the legal justification for accessing data and the extent to which the data contains personal information, various approvals may be required. These can include approvals from local Information Governance and R&D, as well as from local and national Research Ethics Committees. There is usually a requirement for patients to have the option to opt out of any research database. For example, in the UK, the National Data Opt-out^46^ enables patients to opt-out of having their data used for research or planning purposes. Data minimisation and limited storage retention-times are typically required in the case of research databases, as well as precise specification of data-points to be collected.

### Data anonymisation

The DICOM standard (part 15^10^ and supplement 142^47^ has defined a list of metadata attributes that require de-identification. The method of anonymisation may depend upon the requirements, *e.g.* the purpose of the anonymised data set and roles of those requiring access to the data set. Highly configurable tools have been implemented to perform anonymisation^48,49^ with a variety of capabilities:

- Non-reversibly mapping data-values through deletion or replacement with a nominal value
- Generalising and aggregating data-values to retain useful information, *e.g*. converting postcodes to local areas for use with Indices of Deprivation, pseudonymising sensitive information with values to uniquely identify cases
- Performing double pseudonymisation for research databases for sharing with third parties, to decrease the risk of re-identification of the patient
- Encrypting data-values to enable re-identification of data for those with the suitable level of authorisation. This allows updating of cases.

Implementation of anonymisation or pseudonymisation is complicated by inconsistent use of DICOM tags by some manufacturers, *e.g*. free-text fields or private tags can unexpectedly contain patient-identifiable information. If image-data are also used, then caution needs to be taken where personal information has been burnt into image pixels. Software for the removal of such burnt-in data exists.^50^

### Data linking, storing and sharing

Data accessible through a PACS client is limited to images and their associated metadata. In order to pull associated clinical data, PACS data must be linked with other hospital systems *e.g*. clinical systems and research databases. This normally requires manual, or at best semi-automated, integration to select clients and pull linked data. PACS systems use a variety of patient identifiers *e.g*. hospital IDs and NHS numbers, resulting in the requirement for bespoke linking and sometimes necessitating the use of additional fields, *e.g*. the patient’s full name.

Extracted data may be stored locally or in the cloud. For local storage, it may be necessary to engage with the hospital IT department to provision and support the necessary infrastructure. For cloud storage, additional data validation is required since it will be transferred outside of the local hospital.

To share data, a platform and processes are required. In this case, cloud-engineering expertise is required to support the appropriate granular identity and access management control. Some lists of commonly accessed databases can be found in the literature.^51,52^

## Discussion of implications and outcomes

A common model at hospital sites and screening centres is for local storage for imaging and associated clinical data. The data are typically stored in separate, unconnected systems and is managed, in the case of imaging data, by the PACS provider. A variety of use-cases exist that inform the requirements for future implementations and deployments of hospital imaging systems as illustrated in Table 1. Particular issues for consideration are:

- Enabling linkage of data across data sets and locations.
- Providing capability for collection of longitudinal data (previous examinations).
- Retaining information-richness in the data; *e.g*. preserving time intervals for longitudinal data sets.
- Supporting selection of cohorts of patients.
- Easing governance and ethical-approvals processes around data ownership, consent, opt-out, required by the regulatory landscape
- Extending standardisation to new dataflows, *e.g*. HL7 data sets, and improving standardisation of existing data protocols, *e.g*. DICOM

### Storing data

#### Centralised storage

A key benefit of a centralised system, in addition to the higher volume of data that could be mined, would be standardisation and indexing of hospital data storage. However, large-scale IT solutions are notoriously difficult to implement, *e.g.* the costly failure of the UK’s 2002 NHS IT scheme to provide a single central patient record system.^53^ Large national projects should therefore be approached with caution and careful consideration.

#### Storage of DICOM headers separately from image

Querying DICOM header information is easier if it is stored separately from the image. This is more traditionally done by researchers who have downloaded bespoke, highly curated extracts of data sets to their own local environments (*e.g.* NCCID,^48^ OPTIMAM^54^).This method can be used for selected cases or as an automatic process for all cases.

### Querying and retrieving DICOM data from PACS

The methods to query DICOM data will vary according to the requirements, *i.e*. for local audit or large-scale research. As already discussed, the vast majority of image and associated metadata is stored in DICOM compliant format in a PACS and this can cause difficulties in extracting data. Querying and retrieving image data from such a PACS can be done in a variety of ways.

#### Direct query on PACS

Querying on PACS can be performed manually via a PACS client. Alternatively, bespoke software or dose management systems can be used to query PACs and store a subset of the metadata in a distinct database. However, these methods all implement standard DICOM workflow and message formats, which can be inappropriate for data extraction. The list of DICOM metadata tags which can be queried and/or returned, as defined by the DICOM Standard Query/Retrieve Information Models (DICOM Part 4, section C.3^10^), is limited and intended for daily workflow rather than audit or research. Query and retrieval cannot easily be performed in bulk and, in order to retrieve all DICOM tags for a data set, typically the whole image including pixel-data must be retrieved making the process non-scalable.^38^

#### DICOM structured report

One solution to enable retrieval of imaging metadata tags is through DICOM structured reports that can contain metadata for particular purposes. There are a variety of reports, however, only the DICOM radiation structured dose report (RSDR) is commonly used in imaging. RSDRs have the benefit of containing additional exposure-related information but without any image data, thus allowing faster transfer. However, they do not necessarily contain all DICOM metadata of interest from the original images, and may require additional configuration of PACS and imaging modalities.

The use of structured reports also has scope to help further standardise the reporting and storage of clinical reporting^55^ and results but has not been widely adopted.^44^ This is an issue as within the same organisation different records are stored on different software systems. There is often no easy way to link them up. Nationally, the problem becomes even more pronounced with many different systems storing different information in different formats for the same nominal input data.

### Sharing data for research

There are multiple approaches to sharing clinical data and image metadata. A review on Health Data for research^9^ proposes the use of standardised, secure and scalable *Trusted Research Environments* (TRE). These enable access to curated, de-identified data sets for analysis and research with tools for image collection and curation made available as open-source. Approved researchers’ models can then be brought to the data and the TREs themselves can incorporate tools for data-analysis and safe export of results in approved formats. Already a range of data sets exist on the NHS Digital TRE for England, however, none of these currently store DICOM or DICOM-derived data. Open-source access to data-extraction and data-curation code, vendor-neutrality and live-updates to data are clear benefits to storing DICOM metadata in such TRE platforms. By bringing researchers' analysis tools to the data, rather than distributing copies of data, TREs enable access to data whilst minimising risks in de-identification. De-identification can be more robust since barriers to viewing disclosive data and appropriate activity logging can be implemented as integral to the TRE. Any errors in de-identification can be immediately corrected without the need for recalling data. Equally, data attributes that are found to be of value can be subsequently de-identified on the TRE.

In order to enable access to DICOM metadata outside of the hospital PACS environment for local researchers and analysts, or to share research data sets externally the following must be considered:

### Data standardisation and curation

Standardisation is key to making data more accessible and useful.^56^ For example, as previously discussed, the wide range of examination and protocol names in use for the same clinical investigation can make it difficult to group or categorise data in a useful way. One possible solution is to map local names and terminology to a standardised dictionary such as SNOMED.^57^ Although this may involve a significant initial outlay of time it is likely to improve the utility of the data collected.

Data curation is a less simple issue to resolve. Many errors could occur and propagate within data set creation from selection of the wrong procedure name to typographical errors in patient information. A single set of curation solutions are unlikely to resolve all issues. Simple checks can be automated such as identifying impossible or infeasible values for parameters, though these may need to be configured with domain-specific knowledge. It is likely that dedicated curation will still be necessary for research data sets and that periodic audit would be beneficial for all uses.

### Implementation

There are software solutions that can be used to extract data prospectively.^40^ For dose-data collection, both commercial software systems and open-source software, *e.g.* OPENREM,^58^ are available. The Royal Surrey NHS Trust has demonstrated the feasibility of using in-house developed software, through multicentre deployments of its SMART-box^48,59^ and DoseMonitor software systems. These solutions provide a relatively simple way to extract data from PACS both retrospectively and prospectively.

These types of systems can be set up to collect part or all of the image metadata as well as the images themselves. The choice of data-points to be collected must be made carefully, balancing what is reasonably required with ensuring patient confidentiality. However, it must be considered that any data not collected during this process may be difficult to collect at a later date.

Table 2 summarises the salient characteristics of the various models for extraction of DICOM metadata.

**Table 2.** Characteristics of data-extraction models

Data extraction model	Mechanism for retrieval from source storage	Destination storage	Open-source/ proprietary/ bespoke	Case selection	Storage format	Ease of sharing
Traditional PACS-RIS hospital system^39^	Manual pull from PACS initiated from PACS client	-	Proprietary	Retrospective, manual query of limited list of DICOM tags	DICOM	Difficult. Case by case manual download of datasets followed by offline de-identification etc
Extraction to VNA (*e.g., XNAT*,^60,61^ *DCM4CHEE* ^62^)	Automated push from PACS to DICOM store	VNA, *XNAT* or similar	Open-source	Prospective, limited to appropriate PACS forwarding rules	DICOM	Difficult. Case by case download of datasets followed by offline de-identification etc
DICOM storage in Cloud (*e.g.,* Google Cloud Healthcare API^63^)	Push from PACS	Cloud	Proprietary	Prospective, limited to appropriate PACS forwarding rules	DICOM with de-identified export to BigQuery tables	Fully flexible.
Research database^54,64^	Pull from PACS via image collection tools	Local and cloud-based file and database storage	Bespoke, in-house	List of previously selected, retrospective cases.	Flat-files or structured database	Fully flexible.
DICOM metadata extraction^65^	Push from PACS to DICOM listener	Local and cloud-based file and database storage	Bespoke, in-house	Prospective, limited to appropriate PACS forwarding rules and additional	Flat-files or structured database	Fully flexible,

API, Application Programming Interface; DICOM, Digital Imaging and Communications in Medicine; PACS, Picture Archiving Communication System; RIS, Radiology Information Systems; VNA, Vendor Neutral Archive.

## Conclusions

The ability to perform audits using data from PACS has been demonstrated, and implementing changes based on such audits will improve the quality of imaging and workflow. Currently, clinicians, physicists and technicians within a Radiology department have limited access to data from PACS and retrieving that data can be very time-consuming. Whilst some centres have set up automated processes to pull data, these solutions rely either on costly commercial systems or access to staff with considerable computing skills to deploy bespoke software.

Ultimately, the data access process will be easier if it is specified during the PACS procurement process. However, methods laid out in this review, such as structured reports and TREs for researchers, should aid data access facilitating new auditing and imaging research that will be of real clinical benefit to patients.
